# Automated Mango Variety Classification Using Deep Feature Extraction and Machine Learning Classifier Integration

**DOI:** 10.3390/foods15030414

**Published:** 2026-01-23

**Authors:** Ibrar Ahmad, Aftab Khaliq, Bushra Siddique, Mostafa Gouda, Ting Huang, Jinxian Tao, Zhengjun Qiu

**Affiliations:** 1 College of Biosystems Engineering and Food Science, Zhejiang University, Hangzhou 310058, China; ibrarrai@zju.edu.cn (I.A.); mostafa-gouda@zju.edu.cn (M.G.); t3huang@zju.edu.cn (T.H.);; 2 Agricultural Engineering Institute (AEI), National Agricultural Research Centre (NARC) Pakistan Agricultural Research Council, Islamabad 44000, Pakistan; 3 Department of Nutrition & Food Science, National Research Centre, Dokki, Giza 12622, Egypt

**Keywords:** artificial intelligence, transfer learning, machine learning, post-harvest operations, precision agriculture

## Abstract

Manual mango variety classification is time-consuming, error-prone, and contributes significantly to post-harvest losses in developing economies. This study aims to develop a computationally efficient and highly accurate artificial intelligence framework for automated mango variety classification suitable for real-time applications. Eight deep transfer learning models were evaluated as feature extractors and combined with ten classical machine-learning classifiers. Model performance was assessed using accuracy, log loss, memory usage, training time, and inference latency. The hybrid models EfficientNetB0–Linear Discriminant Analysis (LDA) and ResNet50–Logistic Regression achieved 100% test accuracy while reducing inference time by up to 330 times compared to full Convolutional Neural Network (CNN) models. These findings demonstrate that hybrid deep-learning and machine-learning architectures can deliver state-of-the-art accuracy with substantially lower computational cost. Future research will focus on large-scale real-world validation and embedded hardware deployment for industrial fruit sorting systems.

## 1. Introduction

Mango (*Mangifera indica* L.), a member of the Anacardiaceae family, is globally renowned as the “King of Fruits” due to its sweet taste and high nutritional value [1]. There are more than a thousand mango varieties available in countries such as Pakistan, India, and China, each having distinct characteristics in terms of texture (shape, size), color, and aroma [2]. The high demand for mangoes in the international market depends upon the quality of grading and certified packaging [3]. The mango industry is facing significant challenges in the standardization of grading procedures, proper packaging, and certification, mainly because it relies heavily on human labor, which adversely affects the exports [4]. Traditionally, the way of harvesting, grading according to the physical appearance, and also the packaging of mangos is labor-intensive and incompatible with man’s daily working capacity, which leads to fatigue [5]. During post-harvest management, nearly 30–35% of mangos are wasted because of incorrect and delayed identification, grading, and sorting by unskilled labor [6,7]. Therefore, the development and implementation of efficient and intelligent automated systems for mango classification and grading are imperative to meet export quality standards and satisfy the demand for freshly harvested mangos.

Over the Past five decades, artificial intelligence (AI) and computer vision (CV) have significantly influenced the Agri-tech industry, offering a solution to accelerate the processes of fruit harvesting, classification, and grading for quality control and packing [8,9,10,11,12]. The identification of distinct mango varieties presents a particular challenge due to their diverse geometric shapes, forms, and features [13,14]. The scarcity of research literature on mango variety classification, compared to studies on other fruits, is mainly due to the limited availability of relevant datasets. By integrating deep learning (DL) and machine learning (ML) techniques, Computer vision has enabled the recognition of mango types and the evaluation of their quality. Deep learning is a subtype of machine learning technology that uses various layers of neural networks to convert unstructured data into meaningful and valuable insights [15].

Several classification techniques were adopted by the previous research works to ensure the quality of mangos, such as near-infrared spectroscopy and Convolutional Neural Networking (CNN) integration [16] and deep learning-based classification [17]. Deep learning fruit classification methods are widely used in the post-harvest stage and the fruit industry. A research study was conducted to improve mango classification and grading using image processing. The Genetic Algorithm Neuro-Fuzzy Inference System (GANFIS) method was applied to the combined genetic algorithms and an adaptive neuro-fuzzy inference system in feature selection and classification in terms of color, shape, and texture. The outcome of the study indicated high performance with 99.18% accuracy, 98.05% sensitivity, and 97.39% specificity [18]. Pratap et al. proposed a lightweight CNN model for mango classification, achieving 97.50% accuracy trained on a dataset of 1600 preprocessed images comprising 800, 350, and 450 samples across three classes [19]. To improve the classification of mango varieties the MobileNet and hybrid ResNet50 models were tested with data augmentation and principal component analysis (PCA), in combination with different machine learning classifiers. The suggested method identified a 100 percent accuracy, precision, recall, and F1-score, which showed its efficiency [20]. Furthermore, a study employing three CNN architectures VGG16, ResNet152, and Inception v3 for the classification and grading of mango cultivars reported that Inception v3 achieved a classification accuracy of 99.2% and a grading accuracy of 96.7% [7]. The study aims to grade mangoes based on shape and estimate weight using visible imaging. A Fourier descriptor method with Discriminant Analysis (DA) and Support Vector Machine (SVM) achieved 98.3% and 100% accuracy, respectively, while a cylinder approximation method predicted fruit weight with 94.0% R^2^ and 95% grading accuracy [1]. To improve the accuracy of mango grading by means of a multi-level automated system, the ripening stage (through CNN) with 93.23% accuracy, and defects were classified by using a Random Forest model with 95.11% accurcay [21]. Widiyanto et al. applied three grades of mangos, super, A, and B, for the Computer Vision-based classification using Gray-Level Co-occurrence Matrix (GLCM texture-like features and K-Nearest Neighbors (kNN) classification algorithm and kNN achieved an accuracy of 88.88% with a k = 9 value. [22].

Supekar et al. developed a grading system for Dasheri and Kesar mangoes that extracted image features for a Random Forest classifier, applied K-means clustering to segment defects, and used formula-based rules to assign a final grade with 88.88% accuracy [23]. Pandey et al. performed the classification of 15 mango varieties through a transfer learning models (AlexNet, GoogLeNet, ResNet50, VGG16). and by utilizing resized images of 277 × 277 the average performance of the models was measured by calculating the mean, minimum, and maximum values of the F1 score and False Positive Rate (FPR) [24]. Research on the sorting, grading, and classification of mango varieties has employed selected deep transfer learning models, machine learning classifiers, and fine-tuning techniques to identify models’ capabilities to distinguish between different mango types. However, these studies lack clear matrices related to the testing procedures and selection parameters (like training time, inference time of image processing, peak memory usage, and average feature extraction time), which demonstrate the model’s suitability for the dataset. Current methods have not achieved 100% accuracy in the classification of mango varieties. Furthermore, a comprehensive comparative analysis of the integration of deep transfer learning and machine learning for developing fast and accurate models has not been conducted.

Despite significant advances in deep learning for fruit classification, there remains a lack of reproducible, computationally efficient, and real-time capable hybrid frameworks that systematically integrate deep feature extraction with lightweight machine-learning classifiers. Therefore, the scientific problem addressed in this study is the absence of a scalable and low-latency AI architecture for mango variety classification suitable for practical deployment. The aim of this research is to compare eight transfer learning models, integrate the best-performing feature extractors with ten machine-learning classifiers, and identify an optimal hybrid architecture for accurate and real-time mango variety classification.

## 2. Materials and Methods

Initially, in evaluating the proposed methodology against the traditional benchmark method for classifying mango varieties, the manual assessment of mango cultivar classification was considered. Subsequently, a state-of-the-art dual-phase approach was developed for recommending the most suitable machine vision based on an artificial intelligence model for the automated, real-time on-farm classification of different mango varieties. This study utilized a publicly accessible dataset comprising eight mango varieties: Anwar Ratool (AR), Chaunsa (Black) (CB), Chaunsa (Summer Bahisht) (CSB), Chaunsa (White) (CW), Dosehri (DR), Fajri (FR), Langra (LR), and Sindhri (SR). Initially, eight deep transfer learning models, DenseNet201, EfficientNetB0, InceptionV3, MobileNetV2, NASNet, ResNet50, VGG16, and Xception, were assessed through comparative analysis using model performance metrics. Based on criteria such as training time, inference time per image, and peak memory usage, four models, EfficientNetB0, MobileNetV2, NASNet, and ResNet50, were selected for further optimization. In the second stage, the dense layers of these models were removed, and ten machine learning classifiers (SVM, k-NN, Decision Tree, Random Forest, Logistic Regression, Linear Discriminant Analysis (LDA), Quadratic Discriminant Analysis (QDA), Naive Bayes, XGBoost, and LightGBM) were integrated to enhance classification performance. Based on evaluation metrics including Accuracy, Precision, Recall, F1-Score, Log Loss, training time, inference time, and memory usage, EfficientNetB0-LDA and ResNet50-Logistic Regression were recommended for the real-time automatic classification of mango varieties.

### 2.1. Human Assessment of Mango Cultivars

**Figure 1:**
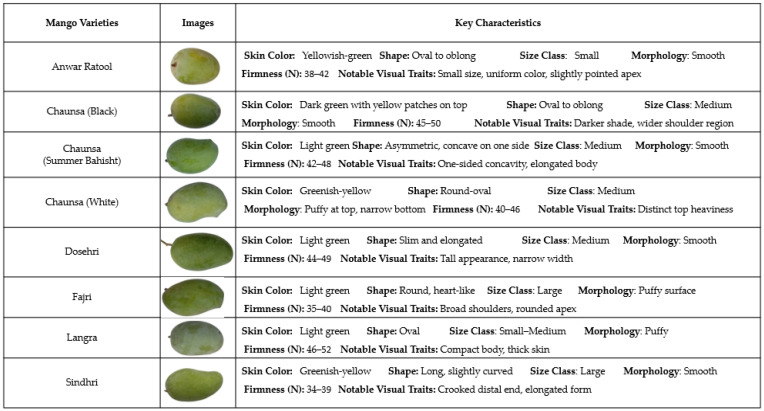
Key characteristics of the samples assessed by individuals who performed the manual classification of the mangoes.

While assessing human appreciation of mango cultivators, eight different varieties of mango, specifically, Anwar Ratool, Chaunsa (Black), Chaunsa (Summer Bahisht), Chaunsa (White), Dosehri, Fajri, Langra, and Sindhri were selected. The samples of each variety taken into consideration were 100, and five people of the mango harvesting workforce were randomly selected. Three farms were selected, located in Multan, Pakistan, and three replications were tested, meaning that 2400 samples of mango were evaluated. The basis of classifications was carried out on visual and tactile inspection of cultivar characteristics, which are specific to such structures as skin, shape, and color. Figure 1 illustrates the key visual characteristics of mango varieties that play a significant role in manual classification. The assessment, with each evaluator separately recording its own classifications without viewing the actual labels and majority voting to obtain the results, was performed. Accuracy, precision, recall, and F-score were also calculated using the counts of true positives as well as false positives and false negatives, with each measurement being computed per cultivar and across replications. The confidence was determined according to the degree of consistency between the assessors (e.g., High in case 4 out of 5 assessors agreed).

### 2.2. Existing Manual Post-Harvest Activities

**Figure 2:**
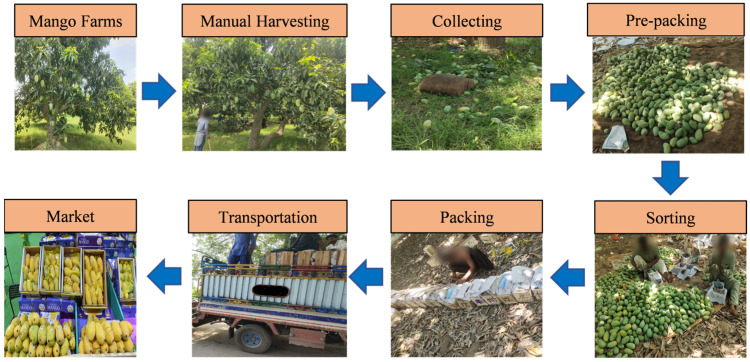
Flow diagram of mango post-harvest activities.

In Pakistan, various processes are generally undertaken in the post-harvest management of mangoes before the fruit reaches both domestic and international markets. At first, mangoes are manually picked from the fields and then brought together at a designated location. The subsequent crucial step involves sorting the mangoes into different varieties. Following this, the same varieties are graded according to quality parameters. Figure 2 presents a detailed flowchart that outlines the post-harvest processes employed by small-scale mango farmers in Pakistan. In most mango supply chains, especially in those with smallholders, different species of mangoes are grown interspersed. These varieties tend to become mixed during harvest as a result of manual labor, unskilled labor, and limited space. This has the potential to cause contaminated batches to be shipped to packhouses where manual separation is prone to error. The accurate classification and grading of mangoes are crucial for reducing the duration required for their transportation from the farm to the marketplace, while also effectively managing post-harvest losses.

### 2.3. Data Acquisition and Preprocessing

**Figure 3:**
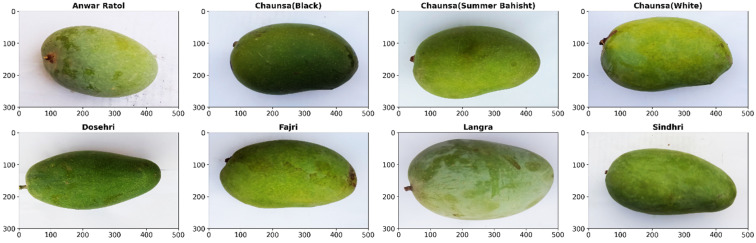
Sample images of the eight distinct mango varieties selected randomly.

This study utilized a publicly available dataset featuring eight varieties of mangoes, namely Anwar Ratool, Chaunsa (Black), Chaunsa (Summer Bahisht), Chaunsa (White), Dosehri, Fajri, Langra, and Sindhri, which are predominantly grown in Pakistan [25]. Figure 3 presents the sample images of the eight distinct mango varieties selected randomly. The dataset comprises a total of 1600 RGB images with a depth of 24 bits, with each category containing 200 images. To enhance model performance and mitigate overfitting, all input images were resized to a fixed dimension of 224 × 224 pixels. Throughout this preprocessing step, the original aspect ratio of each image was preserved to prevent geometric distortion. To achieve methodological purity in a controlled comparative study, data augmentation was deliberately omitted so that the differences in performance between the eight deep transfer learning models can be attributed to their intrinsic architectural design and knowledge pre-training, rather than to the differences and improvements in the training data pipeline. To achieve consistent outcomes, the dataset was split into 60% for training, 20% for validation, and 20% for the final test set. This approach was utilized to assess the performance of the deep transfer models on a real dataset for comparative analysis. Secondly, this facilitated the evaluation of the robustness and generalization abilities of pre-trained models, particularly in the context of small datasets and real-world applications. Finally, this approach mitigates the biases that may arise from data manipulation, thereby allowing for an accurate understanding of the models’ true performance and providing valuable insights for mango variety classification using artificial intelligence. Table 1 represents the distribution of the training, test, and validation sets.

### 2.4. Development of Hybrid Models

The development of a robust artificial intelligence model was accomplished through a two-phase methodology. In the first phase, we evaluated eight deep transfer learning convolutional neural network (CNN) models (DenseNet201, EfficientNetB0, InceptionV3, MobileNetV2, NASNet, ResNet50, VGG16, and Xception), which are frequently utilized for fruit classification tasks. The respective model was evaluated using standard performance metrics, including accuracy, precision, recall, F1-score, log loss, training time, inference time per image, and peak memory usage. Based on this comprehensive evaluation, four models, EfficientNetB0, MobileNetV2, NASNet, and ResNet50, were selected for further optimization, demonstrating a good balance between classification performance and computational efficiency.

**Figure 4:**
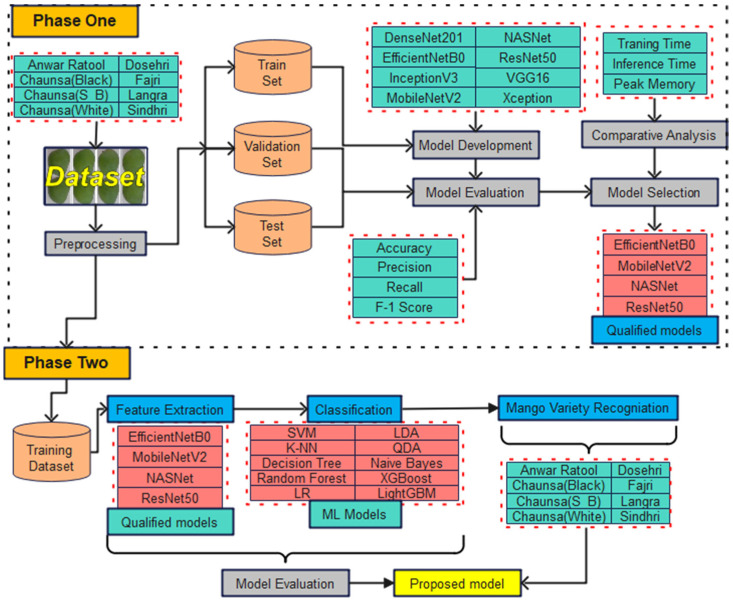
Methodology of the proposed approach.

During the second phase, the dense (fully connected) layers of the selected CNN models were removed, allowing the model to function solely as high-level feature extraction. The feature was subsequently classified using ten conventional machine learning classifiers by predominantly utilizing default hyperparameters to establish baseline performance. Support Vector Machine (SVM)with radial basis function kernel, k-Nearest Neighbors (k-NN, k = 5), Decision Tree using Gini impurity criterion, Random Forest with 100 estimators, Logistic Regression with increased maximum iterations (1000) to ensure convergence, Linear Discriminant Analysis (LDA), Quadratic Discriminant Analysis (QDA), Naive Bayes, XGBoost optimized with logarithmic loss evaluation, and LightGBM employing gradient boosting with histogram-based learning. All the classifiers were trained with the extracted feature vectors and were tested on the test set with extensive measures such as accuracy, weighted precision, recall, F1-score, and log loss of probabilistic prediction. The efficiency of computations was evaluated by measuring the latency of feature extraction, training time, per-image inference time, and memory at its peak. All models are systematically logged by performance metrics, which were then exported to standardized Excel files, allowing direct comparison of all traditional machine learning, ensemble, and discriminative models in a reproducible experimental structure. Through comprehensive experimentation, two optimal combinations of models (EfficientNetB0 and ResNet50) were determined. Subsequently, the pre-trained model that demonstrated the highest performance and satisfied the evaluation criteria was further refined by incorporating a machine learning classifier. These evaluated models functioned as high-level feature extractors, while the machine learning models served as classification models. Figure 4 explains the development of an artificial intelligence model for mango cultivar classification.

### 2.5. Hybrid Efficient Model Development

The proposed system implies the use of the EfficientNetB0 as the primary deep-learning feature extractor, as it has an extensively balanced architecture and constructs outstanding performance when used in transfer learning tasks. EfficientNetB0 operates on inputs of 224 × 224 × 3 and has a sequence of MBConv (Mobile Inverted Bottleneck Convolution) layers of receptive fields 3 × 3, which extract meaningful and efficient features. Its compound scaling method ensures computational and memory efficiency, and it achieves high accuracy (minimizing depth, width, and resolution) through fixed coefficients. The assessment of this characteristic makes EfficientNetB0 a very beneficial tool in real-time low-resource procedures, including automatic fruit classification.

**Figure 5:**
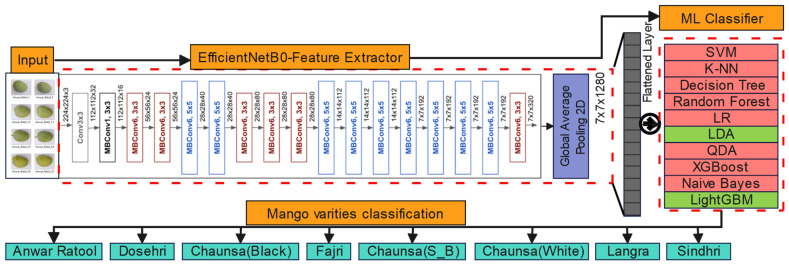
Integration of EfficientNetB0 as feature extractor and ensemble decision networks.

During the next step of the study, the EfficientNetB0 feature vector used as an input to different machine learning classifiers was calculated using the 1280-dimensional Global Average Pooling (GAP). Linear Discriminant Analysis (LDA) and LightGBM were the most outstanding performers among them. A statistical classifier called LDA was used to discriminate the eight mango varieties since LDA increases the class separability through a linear projection. The LightGBM framework is a gradient boosting platform that is fast and accurate, and it proved to be quite efficient in the classification of the mango varieties. Notably, both the EfficientNetB0-LDA model and EfficientNetB0-LightGBM had perfect test classification accuracy of 100% as well as a fast inference rate with minimal memory requirements. Figure 5 demonstrates the classification framework that is based on integrating EfficientNetB0 with other machine learning models. Table 2 describes hardware configurations and hyperparameters of the classifiers applied in the presented study.

### 2.6. Performance Evaluation Metrics

Model evaluation was the initial phase of this research, which took into consideration accuracy, precision, recall, F1-score, and log loss. Finally, the optimization criteria of model selection were chosen based on training time, inference time, and maximum memory utilization. These evaluation parameters and model selection metrics were applied in both stages of model development to the real-time on-farm classification of various mango varieties. Equations (1)–(5) have been used to show the performance evaluation metrics.

(1) $$\mathrm{Accuracy}=\frac{TP+TN}{TP+TN+FP+FN}$$

(2) $$\mathrm{Precision}=\frac{TP}{TP+FP}$$

(3) $$\mathrm{Recall}=\frac{TP}{TP+FN}$$

(4) $$F1=2\times \frac{\mathrm{Precision}\times \mathrm{recall}}{\mathrm{Precision}+\mathrm{recall}}$$

(5) $$Matthews\;Correlation\;Coefficient\;(MCC)=\frac{\left(TP+TN\right)-(FP\times FN)}{\sqrt{\left(TP+FP\right)(TP+FN)(TN+FP)(TN+FN)}}$$

(6) $$\mathrm{Log}\mathrm{Loss}=-\frac{1}{N}\sum_{i=1}^{N}\sum_{j=1}^{8}y_{i,j}\cdot \log(p_{i,j})$$

where

TP = True Positives
TN = True Negatives
FP = False Positives
FN = False Negatives
N: Total number of sample images
*j*: Index of the for each of the mango classes
*y_i_*_,*j*_: 1 if sample i belongs to mango class j; otherwise, 0
*p_i_*_,*j*_: Predicted probability that sample i belongs to class j.

## 3. Results and Discussion

### 3.1. Results of Manual Assessment of Mango Cultivars

The human assessment showed significant variations in classification performance across the different mango cultivars, as shown in Table 3. Chaunsa (White) and Langra provided the greatest accuracy levels of 0.960 ± 0.026 and 0.947 ± 0.021, respectively, which can be explained by their specific morphological features. On the other hand, the lowest accuracy was recorded using Dosehri (0.823 ± 0.040), probably because it was similar to Anwar Ratool. The levels of precision differed significantly, where Fajri scored almost perfectly (0.968 ± 0.027), and Sindhri scored significantly lower (0.756 ± 0.013), meaning that Sindhri was often misclassified as different cultivars. Recall values tracked the accuracy trends, indicating that the high and wild cultivars were consistent among those cultivars that share high accuracy. Overall, the F1-scores used to balance precision and recall were sequentially high (>0.89) in all but the Sindhri cultivar (0.838 ± 0.014), indicating its peculiarities. The low standard deviations in F1-scores (e.g., 0.004 of Langra) imply a high replicability among the evaluators, whereas the large deviations in accuracy (e.g., 0.040 of Dosehri) show the variability between the replications. The results of the findings highlight the significance of morphological obscurity influenced by cultivar, and the ambiguous relationship influences human classification performance with possible implications on quality control in packhouse operations.

### 3.2. Training and Validation

**Figure 6:**
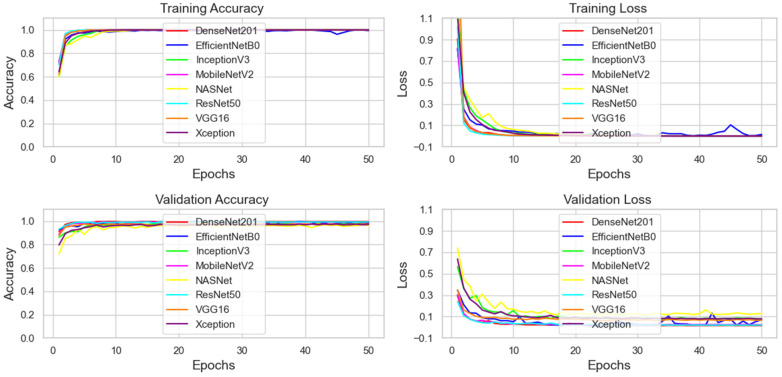
Training of deep transfer learning models.

In the initial phase of the methodology, eight deep learning transfer learning models, DenseNet201, EfficientNetB0, InceptionV3, MobileNetV2, NASNet, ResNet50, VGG16, and Xception, with a SoftMax classifier, were trained and validated to recognize eight different types of mangos. In order to evaluate the learning behavior, the convergence performance, generalization capability, and overfitting tendencies of each model and the training, validation accuracy, and loss curves were plotted against the epochs as shown in Figure 6. The evaluation of these graph curves demonstrates the fact that almost all eight models took a pattern of convergence in which training and validation accuracies approached a value of 1.0. These findings indicate that the selected CNN models demonstrate superior generalization performance along with strong classification ability as far as different mango varieties are concerned. The training and validation loss curves showed a consistent decline and a convergence toward zero, which is an indication of learning well to minimize the errors in classification. It is worth mentioning that the EfficientNetB0 model had certain differences in its learning trajectory as compared to the other models. The accuracy curves’ convergence was close to 1.0, and the loss curve was close to zero; this occurred after most of the models signified successful training, and the features were effectively extracted from the mango images. It means that the method of transfer learning based on pre-trained models succeeded rather well in adapting to the mango classification task.

### 3.3. Comparative Analysis Based on Performance Metrics

Table 4 represents the results of the performance metrics of eight CNN models used in the classification of mango varieties. In all models, the accuracy was high across the training, validation, and test datasets. The overall performance was better in DenseNet201 and ResNet50, with test accuracy, precision, recall, and F1-score being equal to 0.99. These models also had the lowest log loss of 0.02, which showed that they have a high level of confidence in their predictions. EfficientNetB0, MobileNetV2, VGG16, and Xception also showed robust performance with test accuracies dropping to 0.98 and 0.99, alongside the high precision, recall, and F1-scores. InceptionV3 and NASNet received marginally minimal accuracies on test, 0.96 and 0.95, respectively, but the overall performance parameters were high. The training accuracy of all the models was nearly perfect (1.00), and validation accuracies varied between 0.96 and 1.00. The results showed the effectiveness of using transfer learning based on pre-trained CNN models in the mango classification. The efficient performance recorded at all architectures also highlights the strong feature extraction aspects relevant in this task. Overall, the identified results point out the potential of CNN models, in terms of precise mango classification implementation, in the practice of practical applications.

### 3.4. Confusion Matrix Analysis

**Figure 7:**
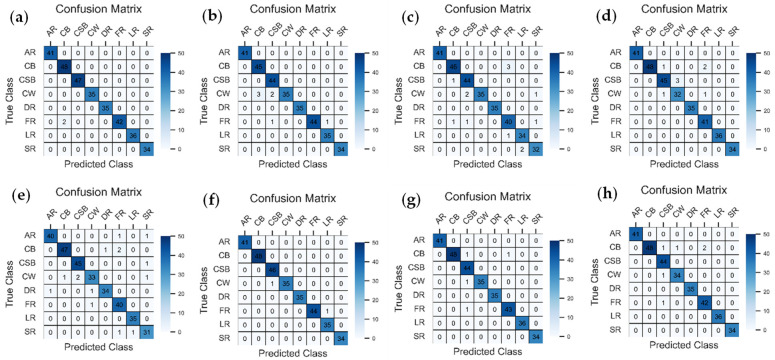
Confusion matrices for (**a**) DenseNet201; (**b**) EfficientNetB0; (**c**) InceptionV3; (**d**) MobileNetV2; (**e**) NASNet; (**f**) ResNet50; (**g**) VGG16; (**h**) Xception.

In order to evaluate the classification performance of the selected deep transfer learning models comprehensively, confusion matrices were created, as shown in Figure 7. In order to allow a more concise presentation of the confusion matrices, the variety name of mangos was abbreviated. In particular, Anwar Ratool, Chaunsa (Black), Chaunsa (Summer Bahisht), Chaunsa (White), Dosehri, Fajri, Langra, and Sindhri were identified as AR, CB, CSB, CW, DR, FR, LR, and SR, respectively. All these confusion matrices were necessary to estimate the performance of models on a class-wise basis and also demonstrate misclassification patterns for each particular model relating to the classification of mango cultivars. Based on the overall confusion matrices, the fact that the various mango varieties were predicted accurately in most of the pre-trained models was mainly attributed to the high number of correct predictions contained within the diagonal boxes. Nevertheless, a few misclassifications were observed even though the confusion matrix indicated high accuracy in class-wise predictions, and these errors were probably a result of the visual similarity of some of the mango varieties.

The DenseNet201 confusion matrix indicated that the model was almost perfect in the classifications of most types of mangoes, with few cases of misclassification recorded between Chaunsa (White) and Fajri. EfficientNetB0 showed a comparable improvement in accuracy at predicting the classes, but Fajri was sometimes confused with Chaunsa (Black). Relating to InceptionV3, there was a general accuracy on each of the predicted classes, but some intersections were noted between the Sindri and Langra classes. Additionally, some cases of misclassification were reported between the Fajri and Chaunsa (Black) categories. After that, the lightweight MobileNetV2 also reported efficient results, but Chaunsa (S_B) was correctly identified as Chaunsa (White), and Fajri was incorrectly identified as Chaunsa (Black) and Chaunsa (White). NASNet exhibited a higher rate of classification errors than many other models; it often confused and misclassified Chaunsa (White) as Fajri and Sindhri and Fajri with Anwar Ratool. Although it could identify the majority of the categories accurately, its ability to differentiate the varieties that are similar in their looks was relatively inefficient. Compared to ResNet50, it was found that this method had an almost perfect classification in all categories. The only mistake was made in distinguishing Langra and Fajri, which reflects on its ability to classify minor characteristics of mangoes. VGG16 had a strong representation in each category, though it was confused with Chaunsa (S_B), Chaunsa (Black), and Fajri. Although the overall classification performance did not change significantly, it was just slightly less accurate than ResNet50 and DenseNet201. Xception has shown really good results with high accuracy in all categories. Nevertheless, the categories that displayed slight misclassifications were Chaunsa(S_B) and Chaunsa (White). The model did well handling intricate features, but it was experiencing minimal challenges in minor deviations of similar classes. In general, the DenseNet201, ResNet50, and EfficientNetB0 performed better in the terms of classification accuracy, but the NASNet faced some difficulties. Chaunsa and Fajri varieties caused classification challenges, unlike Anwar Ratool, Dosehri, and Sindhri, which were successfully classified with high precision by all models.

### 3.5. Model Selection for Optimization

**Figure 8:**
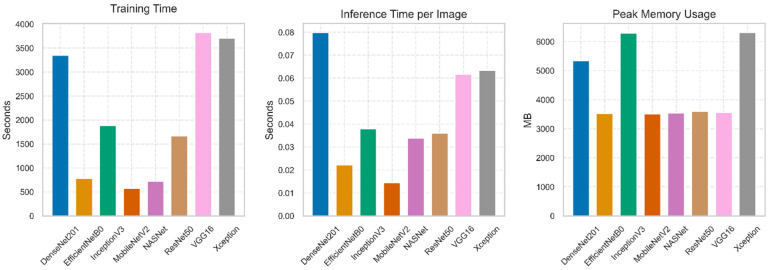
First phase model selection parameters.

The studied deep transfer learning models were compared in terms of selection parameters, including the training time, inference of an image on a computer processor, and the peak memory consumption, which could be optimized in the subsequent processes. The objective of this optimization was to identify the impact of training time on the efficiency of the artificial intelligence (AI) model and the reasons why the inference time of the image is significant in ensuring the real-time recognition of mango varieties. Figure 8 illustrates the output of the comparative analysis of the selected model as per the defined selection parameters. According to the training time, DenseNet201, VGG16, and Xception took relatively more time to train, and thus could not be used for real-time mango variety recognition. Comparatively, the other five models, EfficientNetB0, Inception V3, MobileNetV2, NASNet, and ResNet50, gave satisfactory results, which determined the most suitable models for the next phase of optimization according to their training time. Although when it comes to the responsiveness, the models left out in the first evaluation stage also did not perform optimally in this phase. Simultaneously, the remaining deep transfer learning models demonstrated satisfactory results, indicating their potential for further optimization. them. In the end, the peak memory requirement was observed to be relatively high among the five qualified deep transfer learning models. Inception V3 required relatively high storage space, rendering it unsuitable for real-time mango variety classification. Considering all the selected parameters, it was concluded that EfficientNetB0, MobileNetV2, NASNet, and ResNet50 showed the best results at the first stage, and were permitted to continue for further optimization to make them more efficient.

### 3.6. Comparative Analysis of Hybrid Models

**Figure 9:**
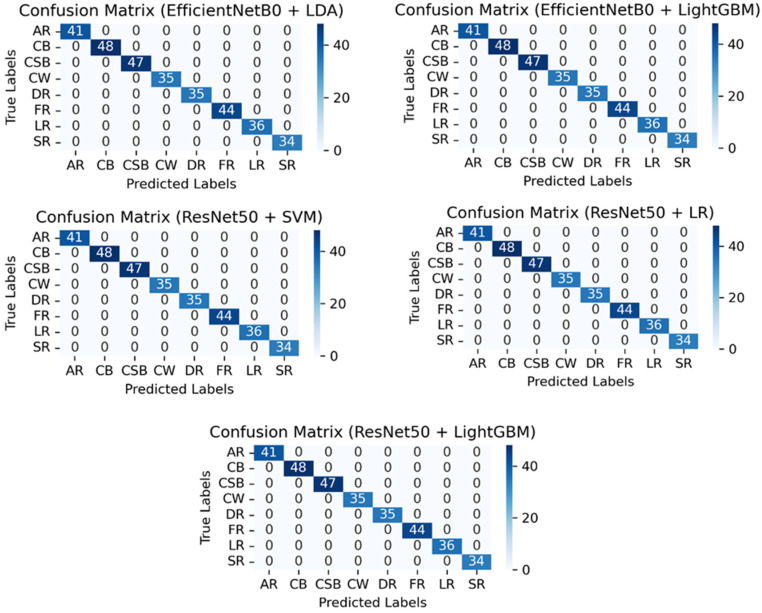
Confusion metrics for high-performance hybrid models.

In the second phase of evaluating four pretrained models such as EfficientNetB0, MobileNet, NASNet, and ResNet50 and nine machine learning classifiers were integrated with each model to optimize first stage qualifier. This has been carried out through determining the metric, including accuracy, precision, recall, F1-score, and log loss. Table 5 and Figure 9 show the overall results of the comparative analysis as measured using the evaluation metrics. The findings showed that the combination of EfficientB0 with LDA and LightGBM made an excellent classification, with the value of precision, recall, and F1-score equal to 1.00, and log loss equal to 0.00. Likewise, ResNet50 combined with SVM, Logistic Regression, and LightGBM presented perfect classification too. During the metrics evaluation, it was noted that machine learning classifiers, including Quadratic Discriminant Analysis (QDA) integration, were not successful in the case of all the pretrained models, and the classification accuracy varied between 0.1688 and 0.23, with the value of log loss being more than 28. Similarly, the Decision Tree classifier also showed poor accuracy, which ranged between 0.6719 and 0.7906 with a maximum log loss of 11.8. The drop in the accuracy of such hybrid models was blamed on overfitting and miscalibration. Based on the high accuracy achieved from the various combinations of deep transfer learning and machine learning classifiers, the following five combinations, EfficientNetB0-LDA, EfficientNetB0-LightGBM, ResNet50-SVM, ResNet50-Logistic Regression, and ResNet50-LightGBM, emerged as the top performers in this optimization phase.

### 3.7. Validation of Top Models: True vs. Predicted Classification

For the comparison of the top five performing hybrid models in terms of validation, the true versus predicted classification in the form of the confusion matrices is illustrated in Figure 9. To validate this process, 320 random test samples were selected from the dataset, and the outputs showed 100% relevancy between the true and the predicted labels, which confirmed the 100 percent accuracy. These hybrid models presented the accuracy and stability of their mango classifications according to varieties, which evidence the deep feature extraction ability of EfficientNetB0 and ResNet50 architecture combined with optimal classifiers. Furthermore, the results of the confusion matrices justify the fitness of these hybrid models, which recorded 100% accuracy in the classification of mango varieties. There are no entries in the off-diagonal elements, i.e., there was no misclassification in all five configurations, which provides the confirmation and utilization of these hybrid models in real-time on-farm modification of different mango varieties.

### 3.8. Interpretation of Grad-CAM Visualizations

**Figure 10:**
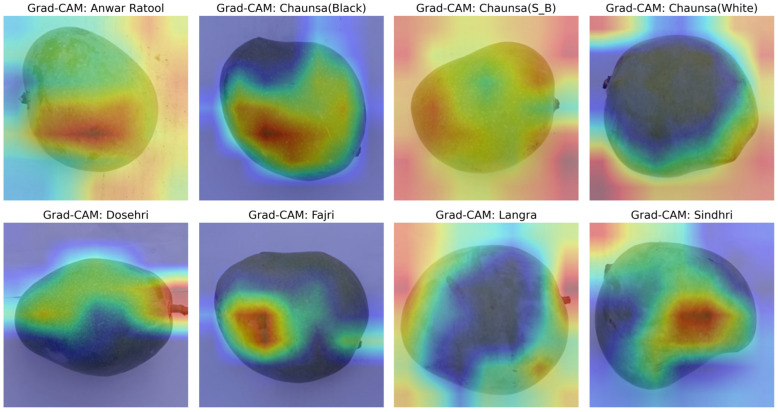
Mango variety-based Grad-CAM attention maps.

Figure 10 illustrates the Grad-CAM attention maps of the eight different mango varieties: Anwar Ratool, Chaunsa (Black), Chaunsa (S_B), Chaunsa (White), Dosehri, Fajri, Langra, and Sindhri. These maps indicate that the trained EfficientB0 model is very successful in allocating the most distinctive regions of the fruit images during the classification. Mostly, the highlighted areas show the middle part of the mango and indicate that the shape, surface texture, and coloring pattern are critical factors in the decision-making process of the model. Particularly, the distinct varieties like Anwar Ratool, Fajri, and Sindhri, the Grad-CAM overlay indicates the focus on the specific patterns or features of surfaces, which highlights the high sensitivity of the model to textural and color signals. On the other hand, the attention patterns in Chaunsa (Black), Chaunsa (S_B), and Chaunsa (White) are more distributed (over the surface), indicating that the model uses global visual features to make the distinction. Correspondingly, in Langra and Dosehri, the model focuses on sections that describe the geometrical shape and surface patterns. The identified results affirm the potential of the deep learning model to effectively learn and generalize the fruit-specific visual characteristics, which indicates a strong and explainable approach to deep automation in the classification of distinct mango varieties.

### 3.9. Final Phase of Model Selection

**Figure 11:**
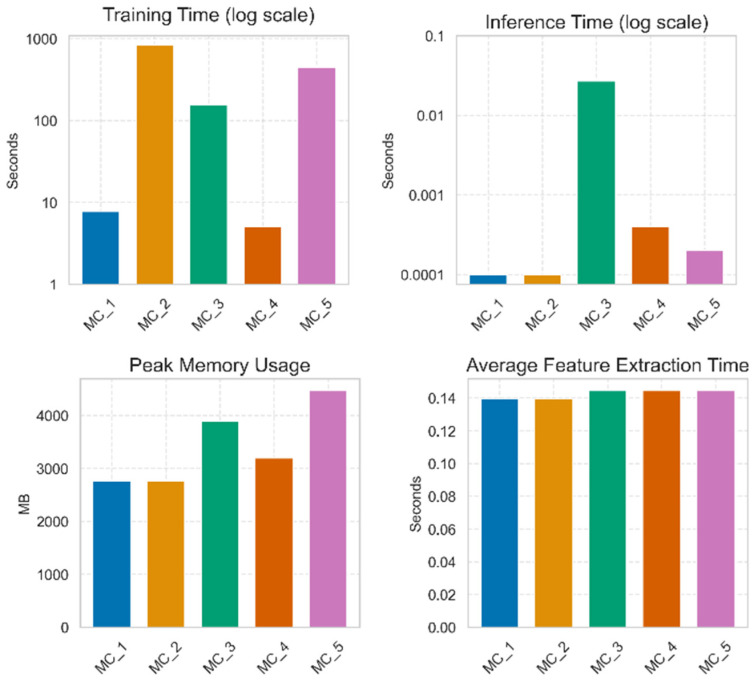
Second phase model selection parameters.

In order to create a reliable and accurate real-time mango variety classification, four key performance indicators were selected: training time, inference time (latency), peak memory usage, and average feature extraction time. Figure 11 represents the comparative example of the top five models according to these performance indicators. Those achieving better clarity and determined visualization of models are referred to in figure as MC_1 to MC_5. The real names of the hybrid models, that are EfficientNetB0-LDA, EfficientNetB0-LightGBM, ResNet50-SVM, ResNet50-Logistic Regression, and ResNet50-LightGBM have been replaced by MC_1, MC_2, MC_3, MC_4, and MC_5, respectively. The training time analysis shows that the following two models, EfficientNetB0-LDA and ResNet50-Logistic Regression, were better to be taken into consideration in terms of training duration than the other three hybrid models. Moreover, EfficientNetB0-LDA and EfficientNetB0-LightGBM were the most appropriate regarding inference time for real-time mango variety recognition. These models were further distinguished as lighter-weight machine learning models sufficient to execute on-farm artificial intelligence-based mango variety classification. The final analyses of evaluation metrics indicate that the average time for extracting the characteristics of all five models is likely to be similar. Therefore, according to shorter inference time and relatively low peak memory consumption, EfficientNetB0-LDA (MC_1) and ResNet50-Logistic Regression (MC_4) are defined as the most suitable options to run in real-time and on-farm mango classification. These models are quite efficient in terms of computational speed and prediction, making them suitable to integrate into a mobile or embedded device, which requires speedy and accurate classification. The comparative analysis of proposed models with their base model is also shown in Table 6.

The empirical results unequivocally establish the superiority of the hybrid models over their monolithic base counterparts. The hybrid paradigm achieved perfect classification efficacy (1.00000) while yielding exponential improvements in computational efficiency. This methodology, which utilizes deep CNNs as frozen feature extractors in conjunction with classical classifiers such as Linear Discriminant Analysis (LDA) and Logistic Regression (LR), reduced training times by two orders of magnitude. For instance, the training time for the ResNet50-LR model was approximately 330 times faster. Furthermore, inference latency was reduced by a factor of up to 221 for the EfficientNetB0-LDA model, accompanied by a significant decrease in peak memory consumption. These substantial efficiency gains are attributable to the decoupling of feature learning from classification. By offloading the identification of the optimal separating hyperplane to highly efficient convex optimization solvers, this approach eliminates the computational overhead associated with end-to-end backpropagation, which is superfluous for tasks where the extracted features are already highly linearly separable.

Table 7 shows a comparative analysis of the current research that carried out assessments and analyzed the sorting and quality of mangoes based on different image processing and machine learning technologies. These studies are, however, not comprehensive, as reflected in the number of varieties classified and the scope of evaluation metrics. Conversely, the proposed study presents a highly scaled solution based on the application of eight deep learning networks and involves an ensemble of eight machine learning classifiers. Additionally, the study also analyzes other important performance indicators, including training time, inference, memory consumption, and feature extraction time, compared to other similar research, which provides a comprehensive benchmark that can be potentially deployed in real-world applications. Generally, the current research makes a substantial contribution to the development of the area because it surpasses the existing research in precision, scalability, and viability levels. From a practical perspective, the suggested framework has a high potential in industrial fruit sorting as it provides a chance to perform automated grading in an accurate and efficient manner, enhance supply chain efficiency, and enforce higher-quality control over exports, which together contribute to the stability of the farmer’s income due to decreased post-harvest losses. In addition to technical and economic advantages, the enhanced classification accuracy also leads to food waste reduction and food security, whereas the focus on scalability and computational efficiency facilitates the overall democratization of AI in agriculture, especially in resource-limited settings.

## 4. Conclusions

This research involved a two-phase approach to selecting machine learning models. In the initial phase, eight distinct machine learning models were trained and assessed. Following the evaluation results, the models that met the criteria were combined with ensemble decision networks to create a hybrid model. This model was developed for an on-farm, real-time automated system to classify mango varieties, aiming to improve post-harvest management of mangoes. In the initial phase of model development, experimental findings revealed that DenseNet201 and ResNet50 achieved the highest test accuracy of 0.99 and the lowest log loss of 0.02. Meanwhile, the test accuracy of the other models also exceeded 0.96, confirming the effectiveness of these deep transfer learning models for real-time classification of mango varieties. After evaluating the training duration, image inference time, and maximum memory consumption as essential factors for a real-time mango classification system, it was determined that EfficientNetB0, MobileNetV2, NASNet, and ResNet50 are suitable for the subsequent optimization phase. Following the outcomes of the second stage of model development, it was determined that EfficientNetB0-LDA, EfficientNetB0-LightGBM, ResNet50-SVM, ResNet50-Logistic Regression, and ResNet50-LightGBM were the leading performers in this optimization phase, achieving 100% accuracy in identifying various mango cultivars. Ultimately, during the second phase, which considered training duration, image inference time, and peak memory usage, it was determined that EfficientNetB0-LDA and ResNet50-Logistic Regression emerged as the top choices for real-time and on-farm mango classification. Analyzing these pivotal findings reveals that the integration of deep learning-based feature extraction with an ensemble decision network has markedly improved the classification accuracy of different mango cultivars. The implementation of this hybrid model in practical scenarios can significantly minimize post-harvest losses and enhance smart post-harvest management through the use of AI in the Food systems. Regarding future prospective projects, we are focusing on the field-scale validation and edge-device optimization.

## Figures and Tables

**Table 1:** Distribution of training, validation, and test sets.

Mango Varieties	Total	Training	Validation	Test
Anwar Ratool	200	120	40	40
Chaunsa (Black)	200	120	40	40
Chaunsa (Summer Bahisht)	200	120	40	40
Chaunsa (White)	200	120	40	40
Dosehri	200	120	40	40
Fajri	200	120	40	40
Langra	200	120	40	40
Sindhri	200	120	40	40

**Table 2:** Specifications of Hardware, Software, and Hyperparameters of Classifiers.

Hardware Unit	Specifications
Central Processing Unit	AMD Ryzen 5 5600 G with Radeon Graphics 3.90 GHz
RAM	32 GB
Graphic Card	NVIDIA GeForce GTX 1060 6 GB
Operating System	Windows 10
Programming Language	Python 3.12
DNN and CNN Framework	TensorFlow 2.19.0
Batch Size	32
Optimizer	Adam
Epochs	50
Loss	Sparse categorical crossentropy

**Table 3:** Human assessment results for different mango cultivars.

Cultivar	Accuracy	Precision	Recall	F1-Score
Anwar Ratool	0.843 ± 0.025 ^de^	0.882 ± 0.031 ^cd^	0.843 ± 0.025 ^de^	0.862 ± 0.026 ^c^
Chaunsa (Black)	0.947 ± 0.032 ^ab^	0.923 ± 0.016 ^ab^	0.947 ± 0.032 ^ab^	0.934 ± 0.011 ^a^
Chaunsa (Summer Bahisht)	0.843 ± 0.031 ^de^	0.944 ± 0.039 ^ab^	0.843 ± 0.031 ^de^	0.891 ± 0.032 ^bc^
Chaunsa (White)	0.960 ± 0.026 ^a^	0.906 ± 0.031 ^bc^	0.960 ± 0.026 ^a^	0.932 ± 0.025 ^a^
Dosehri	0.823 ± 0.040 ^e^	0.929 ± 0.035 ^ab^	0.823 ± 0.040 ^e^	0.873 ± 0.032 ^c^
Fajri	0.880 ± 0.030 ^cd^	0.968 ± 0.027 ^a^	0.880 ± 0.030 ^cd^	0.921 ± 0.011 ^ab^
Langra	0.947 ± 0.021 ^ab^	0.926 ± 0.024 ^ab^	0.947 ± 0.021 ^ab^	0.936 ± 0.004 ^a^
Sindhri	0.940 ± 0.026 ^bc^	0.756 ± 0.013 ^d^	0.940 ± 0.026 ^bc^	0.838 ± 0.014 ^c^

Note: Different superscript letters (^a,b,c,d,e^) within a column indicate statistically significant differences (*p* < 0.05) in the mean metric values between cultivars, based on a post-hoc pairwise comparison test (e.g., Tukey’s HSD) conducted after a one-way ANOVA. Cultivars sharing the same letter within a column are not significantly different from each other. For example, in the Accuracy column, ‘Chaunsa (White)’ (a) is significantly more accurate than ‘Dosehri’ (e), but not significantly different from ‘Chaunsa (Black)’ (ab).

**Table 4:** Comparative analysis of selected deep transfer learning models.

Model	Training Accuracy	Validation Accuracy	Test Accuracy	Precision	Recall	F1-Score	MCC	Log Loss
DenseNet201	1.00	1.00	0.99	0.99	0.99	0.99	0.99	0.02
EfficientNetB0	0.99	0.98	0.98	0.98	0.98	0.98	0.97	0.10
InceptionV3	1.00	0.98	0.96	0.96	0.96	0.96	0.95	0.13
MobileNetV2	1.00	0.99	0.98	0.98	0.98	0.97	0.97	0.05
NASNet	1.00	0.96	0.95	0.95	0.95	0.95	0.95	0.17
ResNet50	1.00	0.99	0.99	0.99	0.99	0.99	0.99	0.02
VGG16	1.00	0.98	0.99	0.99	0.99	0.99	0.99	0.04
Xception	1.00	0.97	0.98	0.98	0.98	0.98	0.98	0.05

**Table 5:** Comparative analysis of selected deep transfer learning models and ensemble decision networks.

Pretrained Model	Classifier	Accuracy	Precision	Recall	F1-Score	Log Loss
EfficientNetB0	SVM	0.9969	0.9969	0.9969	0.9969	0.0578
	k-NN	0.8875	0.8980	0.8875	0.8881	0.4809
	Decision Tree	0.7906	0.7970	0.7906	0.7924	7.5466
	Random Forest	0.9781	0.9782	0.9781	0.9781	0.4883
	Logistic Regression	0.9969	0.9969	0.9969	0.9969	0.0129
	LDA	1.0000	1.0000	1.0000	1.0000	0.0000
	QDA	0.2031	0.2068	0.2031	0.2026	28.7223
	Naive Bayes	0.9125	0.9184	0.9125	0.9131	3.1538
	XGBoost	0.9938	0.9939	0.9938	0.9937	0.0570
	LightGBM	1.0000	1.0000	1.0000	1.0000	0.0086
MobileNet	SVM	0.9938	0.9939	0.9938	0.9937	0.0709
	k-NN	0.95	0.9556	0.95	0.9498	0.2689
	Decision Tree	0.675	0.6804	0.675	0.677	11.7142
	Random Forest	0.9375	0.9401	0.9375	0.9377	0.726
	Logistic Regression	0.9781	0.9785	0.9781	0.9781	0.0456
	LDA	0.9125	0.9181	0.9125	0.9134	0.527
	QDA	0.1688	0.1697	0.1688	0.1664	29.9613
	Naive Bayes	0.9094	0.912	0.9094	0.909	3.2665
	XGBoost	0.9562	0.9571	0.9562	0.9563	0.1467
	LightGBM	0.9594	0.9618	0.9594	0.9598	0.1231
NASNet	SVM	0.9781	0.9785	0.9781	0.978	0.1157
	k-NN	0.8562	0.8811	0.8562	0.8573	1.1947
	Decision Tree	0.6719	0.678	0.6719	0.6716	11.8268
	Random Forest	0.8875	0.8877	0.8875	0.8872	0.6766
	Logistic Regression	0.9719	0.9727	0.9719	0.9716	0.1093
	LDA	0.9562	0.9596	0.9562	0.9564	0.2191
	QDA	0.1938	0.2028	0.1938	0.196	29.0602
	Naive Bayes	0.8062	0.8284	0.8062	0.8062	6.9835
	XGBoost	0.9406	0.9448	0.9406	0.9414	0.2138
	LightGBM	0.9594	0.9603	0.9594	0.9594	0.1474
ResNet50	SVM	1.0000	1.0000	1.0000	1.0000	0.0479
	k-NN	0.9406	0.9454	0.9406	0.9404	0.1451
	Decision Tree	0.7625	0.7636	0.7625	0.7615	8.5604
	Random Forest	0.9844	0.9848	0.9844	0.9844	0.571
	Logistic Regression	1.0000	1.0000	1.0000	1.0000	0.0047
	LDA	0.2	0.1595	0.2	0.1475	12.7539
	QDA	0.1781	0.1892	0.1781	0.1791	29.6234
	Naive Bayes	0.8719	0.8849	0.8719	0.8708	4.6181
	XGBoost	0.9938	0.9939	0.9938	0.9937	0.0557
	LightGBM	1.0000	1.0000	1.0000	1.0000	0.0053

**Table 6:** Comparative analysis of proposed model with base models.

Model	Efficiency	Training Time (s)	Inference Time (s/img)	Peak Memory Usage (MB)
EfficientnetB0 Base Model	0.98438	782.80980	0.02212	3520.43222
EfficientNetB0-LDA	1.00000	7.77000	0.00010	2760.79000
ResNet50 Base Model	0.99375	1669.68638	0.03605	3594.91410
ResNet50-Logistic Regression	1.00000	5.05000	0.00040	3195.31000

**Table 7:** Comparison of the current study with previous studies.

Reference	Objective	Target	Classes	Model	Selection Parameters				Accuracy
					Training Time	Inference Time	Peak Memory Usage	Feature Extraction Time	
[26]	Automated detection of fully/partially ripened mango using machine vision	Harvesting detection for post-harvest management	Mango	Neural Network	×	×	×	×	95%
[27]	Image processing for mango ripening stage detection	Post-harvest Management of Mango	4 mango categories (1.) Green (2.) Ripe (3.) Unripe (4.) Full ripe	RGB and HSV method	×	×	×	×	RGB = 90.4% HSV = 84.2%
[28]	Mango mass grading based on Geometry using image processing	Identification	Harvested Mangos	Faster R-CNN network	×	×	×	×	90%
[21]	Deep grading of mangoes using convolutional neural network and computer vision	Mango maturity classification	Mango	CNN	×	×	×	×	variety recognition 93.23% and quality grading 95.11%
[29]	Mango quality grading using deep learning technique:	Improve agriculture and food industry	Variety of Mangoes	Pretrained Squeeze Net model	×	×	×	×	classification accuracy for RGB images 93.33% and thermal images 92.27% for
[30]	Optimization of mango varieties with transfer learning and deep learning based on classification	-	4 mango varieties	Deep learning—VGG16, Xception	×	×	×	×	Satisfactory
Our Study	Automated Mango Variety Classification Using Deep Feature Extraction and Ensemble Decision Networks	Improve the Export quality of mangos	Eight mango varieties	8 DL Models (DenseNet201, EfficientNetB0, InceptionV3, MobileNetV2, NASNet, ResNet50, VGG16, and Xception) and ML Classifiers	✓	✓	✓	✓	EfficientNetB0 (LDA) = 100% EfficientNetB0 (LightGBM) = 100% ResNet50 (Logistic Regression) = 100% ResNet50 (LightGBM) = 100%

## Data Availability

The image dataset of mango varieties used in this study is publicly available in the Mendeley Data repository, doi:10.17632/5MC3S86982.1. The dataset can be accessed and downloaded directly via the following link: URL (https://data.mendeley.com/datasets/5mc3s86982/1, accessed on 19 January 2026). The dataset and code used to support the findings of this study are available in the GitHub repository at: https://github.com/IbrarRai786/Mango_classification_hybrid_deep_learning, accessed on 19 January 2026.

## Abbreviations

The following abbreviations are used in this manuscript:

AI	Artificial intelligence
LDA	Linear discriminant analysis
CV	Computer vision
DL	Deep learning
ML	Machine learning
CNN	Convolutional Neural Networking
GANFIS	Genetic Algorithm-Adaptive Neuro-Fuzzy Inference System
PCA	Principal component analysis
DA	Discriminant Analysis
SVM	Support vector machine
MCC	Matthews Correlation Coefficient
GLCM	Gray-level co-occurrence matrix
kNN	k-Nearest neighbors
FPR	False positive rate
Grad-CAM	Gradient-weighted class activation mapping
AR	Anwar ratool
CB	Chaunsa (Black)
CSB	Chaunsa (Summer Bahisht)
CW	Chaunsa (White)
DR	Dosehri
FR	Fajri
LR	Langra
SR	Sindhri
QDA	Quadratic discriminant analysis
MBConv	Mobile inverted bottleneck convolution
AM	Activation matrix
Adam	Adaptive moment estimation
